# Undernutrition and Associated Factors among Under-Five Orphan Children in Addis Ababa, Ethiopia, 2020: A Cross-Sectional Study

**DOI:** 10.1155/2021/6728497

**Published:** 2021-11-01

**Authors:** Selam Shegaw Sewnet, Hunegnaw Almaw Derseh, Hanna Demelash Desyibelew, Netsanet Fentahun

**Affiliations:** ^1^Amhara Regional Health Bureau, North Shewa Zone, Siyadebrina Wayu Woreda Health Office, Deneba, Ethiopia; ^2^Department of Nutrition and Dietetics, School of Public Health, College of Medicine and Health Sciences, Bahir Dar University, Bahir Dar, Ethiopia

## Abstract

**Background:**

Undernutrition contributes to the death of around 3 million children and threatens the futures of hundreds of millions, undermining healthy development and the strength of their societies by preventing children from achieving their full potential. Orphans are at greater risk of undernutrition because they are more likely to be extremely poor and receive less medical and social care. However, there is little information about the prevalence of undernutrition and associated factors among under-five orphan children.

**Objective:**

This study aimed to assess undernutrition and associated factors among under-five orphan children in orphanages in Addis Ababa, Ethiopia.

**Methods:**

An institution-based cross-sectional study was conducted in Addis Ababa from February 28 to March 28, 2020. A simple random sampling technique was employed to recruit a total of 275 orphan children. An interviewer-administered questionnaire and anthropometric measurements were used to collect data. Data were entered using EpiData version 3.1 and analysis was done by WHO Anthro version 3.2.2 and SPSS version 23. Multivariable logistic regression analysis was performed to identify determinants of undernutrition at a *p* value of less than 0.05 with an adjusted odds ratio of 95% confidence interval.

**Results:**

The prevalence of wasting, stunting, and underweight were 11.1%, 45.8%, and 25.5%, respectively. Presence of illness (AOR = 2.23; 95% CI: 1.41, 12.73), children who received less than three meals per day (AOR = 2.11; 95% CI: 1.58, 7.71), and children who were not vaccinated (AOR = 2.86; 95% CI: 2.07, 11.61) were significantly associated with stunting. Children who were not vaccinated (AOR = 2.04; 95% CI: 1.29, 9.71) and who had inadequate dietary diversity scores (AOR = 1.32, 95% CI: 1.16, 12.65) were significantly associated with wasting and underweight, respectively.

**Conclusion:**

The prevalence of undernutrition was very high compared to national data. Health status, meal frequency, and vaccination status were associated factors of stunting. Vaccination status and dietary diversity score were associated factors with wasting and underweight, respectively. Therefore, improving meal frequency, dietary diversity, and early treatment during childhood illness are important to reduce orphan undernutrition.

## 1. Introduction

Undernutrition denotes the insufficient intake of energy and nutrients to meet an individual's needs to maintain good health [1]. Malnutrition is affecting a highly vulnerable population in several regions of the world [2]. Under-five children are the most vulnerable age group for undernutrition particularly in developing countries which is among the most serious health problems facing Ethiopia [3]. Undernourished children have lower resistance to infection and are more likely to die from common childhood illnesses such as diarrheal diseases, febrile illness, and respiratory infection [4]. Undernutrition increases the frequency and severity of such infections and contributes to delayed recovery. In addition, the interaction between undernutrition and infection can create a potentially lethal cycle of worsening illness and deteriorating nutritional status [5].

Malnutrition is affecting a highly vulnerable population in several regions of the world. According to the global nutrition report estimation, undernutrition contributes to the death of around 3 million children per year and threatens the futures of hundreds of millions, undermining the healthy development of their bodies and the strength of their societies by preventing children from achieving their full potential [6]. This problem in the early stages of life can increase the risk of infections, morbidity, and mortality together with decreased mental and cognitive development [3].

An estimated 17.8 million orphan children in the world had lost both parents (double orphan) and 153 million children in the world are orphans (single orphan) with more than one in seven children orphaned in Sub-Saharan Africa [1, 7]. In Ethiopia, 5.5 million children (around 6% of the total population) are categorized as orphans or vulnerable children. Orphan comprises almost 12% of Ethiopia's total child population, and half of the children below the age of five are stunted [1, 3].

The children in orphanages had a significantly higher rate of undernutrition than nonorphanages. Consequently, many orphan and vulnerable children are still in a difficult situation and seek immediate attention [6, 8].

Orphaned and institutionalized children may experience one or several micronutrient deficiencies. Children may contract an infection due in part to poor nutritional status and are vulnerable to infections since they are at risk for a variety of complications, putting their health and development in great trouble [1].

Due to poverty, family disintegration, domestic violence, disability, and social unrest, the number of orphans is expected to increase in the next future. These children are most vulnerable and may be at greater risk from child labor, trafficking, prostitution, abduction, stigma, discrimination and they are potentially at greater risk of malnutrition because they are more likely to be extremely poor and receive less medical and social care. This portion of the population seeks immediate support for their survival and growth, despite less number of orphanages compared to the magnitude of orphans and vulnerable children [9, 10].

Orphan children in Ethiopia are the most prevalent forms of social problems and are vulnerable to all forms of abuses and exploitations, loss of inheritance rights, loss of opportunity for education, basic health care, normal growth, and development as well as shelter. And they are also at risk of future incidents of HIV infection. They faced many problems including basic needs such as food, safe water, parental care, supervision, and protection. As a result of this, they suffer from malnutrition and poor health. Children living in orphanages tend to be neglected and become malnourished [10–12].

United Nations international children's fund and World Bank review in New York indicated that the situation of orphans and vulnerable children receives little attention in poverty reduction strategy papers and national strategic plans, despite the large magnitude of the problem that existed in some countries [13]. Based on the National Nutritional program, millions of Ethiopians are still under chronic and acute malnutrition that ranks among the top, both in Sub-Saharan Africa and the world. Although the progress and achievements observed so far can be celebrated, the deep-rooted causes of malnutrition in the country call for high-impact, integrated, and coordinated interventions to end hunger [14].

Another assessment made by the former Children and Youth Affairs Organization in Ethiopia showed the problems faced by the orphanages as inadequate funding to support programs designed for the children, shortage of trained personnel, inadequate skills training that resulted in long care in an orphanage, lack of psychosocial service, and lack of long-term strategic planning.

However, there is a limited study conducted on the nutritional status and associated factors among institutionalized under-five orphaned children in Ethiopia and there is no study conducted in the study area. Thus, information regarding the nutritional status of orphan and vulnerable children and associated factors is limited in the study setting. Therefore, this study will be helpful to fill the gap by providing information for policymakers, NGOs, and other stakeholders.

## 2. Methods and Materials

### 2.1. Study Area

This study was conducted in Addis Ababa which is the capital city of Ethiopia. There are 8 under-five orphanages in Addis Ababa which are given licenses by the Addis Ababa women's and children's affairs office. These orphanages are devoted to the care and raring of children who lost their parents and some of these orphanages give health care services for the people outside the orphanage and give support for the vulnerable and fostered children. There are 406 under-five orphan children reared in orphanages.

### 2.2. Study Design and Period

An institutional-based cross-sectional study was conducted from February 28 to March 28, 2020, in orphanages in Addis Ababa

### 2.3. Study Populations

The study populations of this study were all institutionalized under-five orphan children residing in Addis Ababa orphanages.

### 2.4. Inclusion Criteria and Exclusion Criteria

All under-five orphans who had stayed full-time at the orphanages were included in this study.

### 2.5. Sample Methods and Procedure

The sample size for the prevalence objectives was calculated using single population proportion formula determined by using the prevalence of stunting, wasting, and underweight (35.1%, 7.5%, and 8.9%) with 4%, 2%, and 2% margin of error, respectively, from a similar study conducted in Hawassa town [15] and 95% CI using the formula *n* = (*Z a*/2)^2^*p* (1-*q*)/*d*^2^. By adding a 10% nonresponse rate and by considering the population correction formula, the sample size for the prevalence objectives (stunting = 243), (wasting = 261), and (underweight = 275). Finally, the highest calculated sample size from the three indicators was taken as the final sample size, that is, 275. The sample size was also calculated for the associated factors objective through considering different factors [15–17] which were significantly associated with outcome variable with 95% level of confidence, a ratio of unexposed to exposed 1 : 1, power of 80%, and taking in account 10% nonresponse rate. Finally, by considering the population correction formula, the maximum sample size was found to be 222. The final sample size for the associated factors objective was lower than for the prevalence objectives. Therefore, the maximum sample size from the first specific objective (*n* = 275) was used for this study.

A simple random sampling technique was used to recruit a total of 275 study participants. The sample size required for each orphanage was allocated proportionally to the number of orphans in each orphanage. Participants were selected from each stratum randomly from the list of the orphan children registered in the orphanage administration book.

### 2.6. Data Collection Tools and Measurements

Data were collected by using an interviewer-administered, pretested, and structured questionnaire. The questionnaire was initially prepared in the English language by adapting from different sources. The prepared tool was translated into the Amharic language and again back to English to check its consistency. The questionnaire was composed of sociodemographic characteristics, hygiene, and environmental factors, feeding practice, health-related variables, and anthropometric measurements.

Data were collected by three data collectors and one supervisor after receiving training for two days by principal investigators in the Amharic language about methods of anthropometric measurement, interviewing technique, and filling questionnaires.

*The anthropometric measurements*, height and weight, were measured using a standard measuring scale using standard operating procedures. Weights of under two year's children were measured by spring balance weighing without shoes and in light cloth to the nearest 0.1 kg. Beam balance was used to measure the weight of children above two years. The scale was calibrated immediately before and during each session by placing standard calibration weights of 5 kg iron on the scale to ensure accuracy.

*The length* was measured using a wooden board in recumbent position while the child was barefooted and free of a head wearing on children <2 years to the nearest 0.1 cm and height was measured using a wooden board in standing-up position while the child was barefooted and free of any head wearing on children >2 years and recorded to the nearest 0.1 cm. The child was positioned feet together: feet flat on the ground, heels touching the backplate of the measuring instrument, legs straight, buttocks against the backboard, scapula against the backboard, and arms loosely at their side.

These measurements were compared and classified of nutritional status using WHO standard growth curves for under-five children. Height for age, weight for age, and weight for height were expressed in standard deviation units (Z scores) from the median of the reference population. The use of this reference population is based on the finding that well-nourished young children in all population groups follow very similar growth patterns. The reference populations are useful for comparison facilitating the examination of differences in the anthropometric status of subgroups in a population and changes in nutritional status over time [18].

Dietary diversity score was assessed based on the number of food groups consumed from the seven food groups within the last 24 hours. Accordingly, children were classified as having adequate dietary diversity scores if they had consumed four and more from seven food groups [19].

### 2.7. Data Quality Control

Data quality was assured initially through the careful design of the questionnaire and data collection procedure. The data collectors and supervisor were trained for two days on data collection procedures by developing manuals relevant to achieving the objectives of this study. They were trained on how to approach the study subjects, how to record data, how to measure weight and height, and how to control missing data by the principal investigator. In addition to delivering training, the principal investigator and supervisor discussed how to supervise data collectors. A supervisor supervises the data collectors daily to assure that the data collection activities were carried out according to the training guide.

The questionnaire was prepared originally in English language and then translated into Amharic language and retranslated into English by a language expert. Almost all of the questions were adopted from other previously conducted similar studies with little modifications [12,20–22]. A pretest was employed before engaging to full implementation of data collection by taking 5% of the sample size (*n* = 14) outside the study area.

The responses of participants were checked by supervisors and the principal investigator by administering the questionnaire at the end of data collection to randomly selected 10% of orphans already visited by the data collectors. Moreover, a supervisor was checking everything recorded by data collectors in each questionnaire daily to ensure no data are missing, and as a result, the data were precise and accurate. Data collectors themselves checked for internal consistency, that is, the extent to which the responses to different questions correlate with each other during interviewing each recruited orphan so that they reconfirmed the responses of the interviewees.

### 2.8. Data Analysis

The data were entered, cleaned, and coded using EpiData version 3.1 and then exported to Statistical Package for Social Sciences (SPSS) version 23 for analysis. Anthropometric indices stunting, underweight, and wasting were generated by using WHO Anthro version 3.2.2, and results were classified according to WHO cut-off points. Descriptive statistics, frequency, percentages, table, figure, and mean and standard deviations were used to present the data. Multiple binary logistic logistics regression analyses were used to address the effect of confounders, and a 95% confidence interval was used to estimate the precision of the odds ratio. The assumption of goodness of the model was checked by Hosmer–Lemeshow test (*p* value 0.12, 0.35, and 0.58 for wasting, stunting, and underweight), and multicolinearity was checked using the variance inflation factor. Finally, those variables that showed *p* value <0.05 in multivariable analysis were taken as an independent predictor of undernutrition.

### 2.9. Ethical Consideration

Ethical clearance was obtained from the Ethical Review Board of Bahir Dar University. A formal letter was obtained from Bahir Dar University School of Public Health and was submitted to the management of the orphanages. The directors of the orphanages were consulted before the data collection. Information regarding the aim of the study was explained to the directors, caretakers, and participants, including the procedures, potential risks, and benefits of the study. The respondents were informed that they can refuse not to participate in the study. Confidentiality was by assuring the information was not to be used for another purpose. Written informed consent was required from the orphanage directors and the caretakers, and verbal assent from the children after being informed about the study.

## 3. Results

### 3.1. Sociodemographic Characteristics of Study Participants

A total of 271 under-five orphan children were involved in this study making the response rate of 98.9%. The mean (±SD) age of the children was 27 (±16.31) months and about 163 (60%) of the participants were males. Among all, 90 (33%) of children were in the age category of 36 and above months, and two-thirds 167 (61.6%) of their caretakers were between the age categories of 19 and 35 years. About 159 (58.7%) and 196 (72%) of caregivers were married and educated, respectively. Around 157 (60%) lived for less than five months in the orphanage (Table 1).

### 3.2. Health and Diet-Related Characteristics of Study Participants

About 175 (65%) of under-five orphan children were vaccinated, and of them, only 40 (15%) were fully vaccinated. Ninety-two (34%) of them had experienced an illness in the last two weeks. Fifty-six (61%) and 57 (62%) of children had fever and diarrhea, respectively, in the last two weeks before the data collection. About 30 (33%) of sick children get treatment in the health facility of the orphanage. Related to children's dietary status, 73 (27%) of them had consumed four and more food groups and 81 (30%) had meal frequency of three and more times in the last 24 hours (Table 2).

### 3.3. Hygienic, Sanitation, Care, and Support-Related Characteristics

Regarding caregivers' hygienic practice, the majority 254 (94%) of them had washed their hands whenever they feed the child, and 254 (94%) of them had washed their hands after cleaning a child, but only 23 (9%) of them washed their hand before breastfeeding. About 244 (90%) and 236 (87%) of caregivers had washed their hands before preparing food and after visiting the toilet, respectively. Of those who washed their hands after visiting the toilet, only 34 (13%) washed their hands with water and soap. Regarding sources of water for domestic consumption, about 260 (96%) of them were from a piped water source (Table 3).

### 3.4. Prevalence of Undernutrition (Stunting, Wasting, and Underweight)

**Figure 1:**
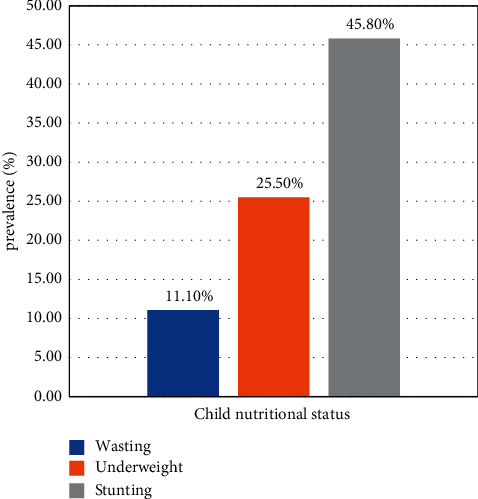
Prevalence of under nutrition among children living in an orphanage in Addis Ababa, Ethiopia, 2020 (*n* = 271).

Among all children taking part in this study (*n* = 271), the prevalence of stunting was 45.8% (95% CI: 39.9, 52), wasting 11.1% (95% CI 7.7–15.1), and underweight 25.5% (95% CI 20.3–31) among orphan children (Figure 1).

### 3.5. Factors Associated with Undernutrition

All independent variables were examined for possible associations with stunting, wasting, and underweight using a binary logistic regression model. A bivariable analysis was first performed to identify predictor variables associated with the outcome variables. Variables with a *p* value <0.2 in the bivariable analysis were fitted into the multivariable analysis to control the possible effects of confounder and to see their independent association with stunting, wasting, and underweight.

### 3.6. Factors Associated with Stunting

Six variables (age of the child, meal frequency, age of the caretaker, treatment, marital status, and vaccination) were associated with stunting during bivariable analysis (*P* value  = <0.2). In the multivariable analysis, treatment, meal frequency, and vaccination were significantly associated with stunting at *P* value <0.05 with 95% CI.

Orphan children who had a history of illness were two times more likely to be stunted (AOR = 2.23; 95% CI: 1.41, 12.73) than those who had no history of illness. The probability of being stunted was two times higher for children who received less than two meals per day (AOR = 2.11; 95% CI: 1.58, 7.71) than those who received three and more meals per day. Similarly, children who were not vaccinated were nearly three times more likely to be stunted (AOR = 2.86; 95% CI: 2.07, 11.61) as compared to those children who were vaccinated (Table 4).

### 3.7. Factors Associated with Wasting

In the bivariable analysis, vaccination received, meal frequency, age of the child, and duration of the stay were associated with wasting. Among those variables, only vaccination status remained independently associated with wasting in the multivariable analysis. The odds of wasting were high among children who were not vaccinated (AOR = 2.04; 95% CI: 1.29, 9.71) than those who were vaccinated (Table 5).

### 3.8. Factors Associated with Underweight

Age category of children, sex of the child, dietary diversity score, water treatment, illness during the last two weeks before the survey were variables with a *p* value of <0.2. Then, all these variables were fitted into the multivariable analysis, and only the dietary diversity score of children was statistically significant with underweight. The likelihood of being underweight was 1.32 times higher among children with low dietary diversity scores (AOR = 1.32, 95% CI: 1.16, 12.65) than those children with adequate dietary diversity scores (Table 6).

## 4. Discussion

This study aimed to assess the prevalence of undernutrition and associated factors among children in orphanages. Accordingly, among all children who have taken part in this study, 11.1%, 45.8%, and 25.5% were wasted, stunted, and underweight, respectively. Lack of treatment, less meal frequency, and lack of vaccination were factors significantly associated with stunting. Likewise, lack of vaccination was associated with wasting, and the associated factor for underweight was a lower dietary diversity score.

The prevalence of wasting, stunting, and underweight in the current study was consistent with a study conducted in Gondar Town, Ethiopia, which reported a wasting prevalence of 10%, underweight of 28%, and stunting of 46% [15] and the regional state of Amhara with wasting prevalence of 9.8%, underweight of 28.4%, and stunting of 46.3% [23] However, the prevalence of stunting and underweight in the current study was higher than that reported from India and Kenya [8, 24]. This discrepancy might be due to the difference in study areas, a lifestyle of institutionalized orphaned children because of different settings in the orphanage, difference in number of orphanages in which in Ethiopia, there are few orphanages that care and rear many orphan children in a single institution, where care and support the children get may not be appropriate and easily vulnerable for infection; these all may lead to children vulnerable to nutritional problems and rise the prevalence of undernourished children. The other possible reason for the difference might be the presence of different dietary patterns and meal menu in the orphanages in which in the current study, most of orphan children's dietary diversity scores and meal frequency were less than the standard recommendations, which are the causes of undernutrition.

The prevalence of wasting in the current study agreed with other study finding in Ethiopia [16]. However, compared to other studies, the prevalence of wasting reported by the current study was lower than from the Somali region, Ethiopia [14]. The reason for this discrepancy might be due to the difference in setting and infrastructure of orphanages; the current study was conducted in Addis Ababa, a capital city of Ethiopia that has better infrastructure, care, and support in the orphanages as compared to the study done in Somali region. Orphanages in Addis Ababa have regularly been checked by the Addis Ababa women's and children affairs office to assure whether or not children's took adequate nutrients according to the menu of the orphanage and got better care and support from the orphanages; this may improve children's dietary intake and health in which these reduce the prevalence of undernourished children.

Stunting was higher among children who had a meal frequency below 3 times per day. This finding is also supported by another research conducted in Aykel town, Ethiopia [9], and Afghanistan [25] in which the findings of both studies showed that most of the children with stunting were those who feed less than three times per day. This might be due to the reason that feeding less frequently for children per day based on their age resulted in inadequate intake of nutrients which is one of the immediate causes of undernutrition. This implied that fulfilling the nutrient requirement of children through age-appropriate feeding frequency is crucial to stunting and other forms of malnutrition.

The probability of the presence of illness increases the odds of developing stunting. Infection increases the loss of nutrients, reduces appetite and intake of food, and finally leads to one form of undernutrition (stunting, wasting, underweight), one of the immediate causes of undernutrition. Our study finding showed that stunting was significantly higher among those children who were not treated for their illness. This study report is supported by studies conducted in Gondar Ethiopia and Kenya. Children who were sick were more likely to be stunted, underweight, and wasted [8, 16].

Children who were not vaccinated were more likely to be stunted and wasted than those who were vaccinated during their childhood period. Our study finding showed that the odds of wasting and stunting were higher among children who were not vaccinated than those who were vaccinated. Similar study findings were reported from Southern Ethiopia and Indonesia both of which indicated being immunized has a decreased risk of being underweight than being nonimmunized [26]. This may be because children lack and/or has weak body defense mechanism which enables them to be infected by several infectious and vaccine-preventable diseases. In turn, not being vaccinated means that children will be exposed to and infected by different infectious agents easily which may be progressed to disease and eventually cause undernutrition.

In this study, dietary diversity score was significantly associated with underweight. The odds of underweight among children with low dietary diversity scores (<4) were 1.32 times higher as compared to children with adequate dietary diversity scores. Evidence from different sources indicated that orphan child nutritional intake was deficient for all nutrients when compared to RDA, depriving them of a balanced diet and causing low dietary diversity score which was a significant predictor of underweight [27, 28]. Similarly, a study done in Sidama Zone, Ethiopia, also reported that dietary inadequacy and low diet quality in terms of diversified diet and availability of micronutrients had a significant association with underweight [27]. This could be partially explained by the fact that consumption of a poor quality diet restricts the physical growth of children.

### 4.1. Limitations of the Study

Since this study was aimed to assess the nutritional status and associated factors among the orphan under-five children by using a cross-sectional study design and due to the cross-sectional nature of the design, it is unable to identify the causal effect relationship between the outcome and independent variables. Since the data were collected at one spot time (cross-sectional), it does not identify the existing undernutrition was developed within the institution or before the entrance of the institution.

The analysis used in this study (logistic regression analysis) has less power of significance in determining associated variables with the outcome variables as compared to other analysis methods. There may be social desirability bias in responding to some dietary assessing questionnaires because the study was conducted in orphanages and the respondents were caretakers/guardians within the institution that may have overestimated the types and frequency of foods given to the children. Since respondents were orphan children, they are more susceptible to psychiatric problems; but this study did not assess the psychiatric factors which is another limitation of this study. Conclusion based on the WHO cut-off point for declaring the public importance of undernutrition: the prevalence of undernutrition was found to be higher among orphan children. History of illness, less meal frequency, and lack of vaccination were factors significantly associated with stunting. Likewise, lack of vaccination and lower dietary diversity score were associated with wasting and being underweight, respectively. Therefore, improving meal frequency, dietary diversity, immunization coverage, and early treatment during childhood illness will be very important to reduce orphan undernutrition.

## Figures and Tables

**Table 1:** Sociodemographic characteristics of study participants living in orphanages, Addis Ababa, Ethiopia, 2020 (*n* = 271).

Variable	Frequency	Percent
*Sex of the child*		
Male	163	60.10
Female	108	39.90

*Age of the child in months*		
6–11	52	19.20
12–23	78	28.80
24–35	51	18.80
≥36	90	33.20

*Age of the caregiver in years*		
<20	10	3.7%
21–45	207	76%
>45	54	20%

*Educational status of the caregiver*		
Uneducated	75	27.7
Educated	196	72.30

*Marital status of the caregiver*		
Unmarried	112	41.3
Married	159	58.70

*Natural parents of the child alive*		
Yes	71	26.20
No	200	73.80

*Is the orphan transferred from another orphanage?*		
Yes	3	1.10
No	268	98.90

**Table 2:** Health and diet-related characteristics of orphaned children living in Addis Ababa, Ethiopia, 2020 (*n* = 271**)**.

Variable	Frequency	Percent
*Received vaccination*		
Yes	175	64.6
No	96	35.4

*Vaccination status*		
Fully vaccinated	67	38.3
Not fully vaccinated	204	75

*Illness in the last two weeks*		
Yes	92	33.9
No	179	66.1

*Diarrhea in the last 2 weeks*		
Yes	57	21
No	214	13

*Vomiting in the last 2 weeks*		
Yes	32	12
No	239	88

*Seek treatment for the illness?*		
Yes	62	23
No	30	32.6

*Dietary diversity score*		
<4 food groups	198	73.1
≥4 food groups	73	26.9

*Meal frequency of children*		
<2 times/day	190	70.2
≥3 times/day	81	29.8

**Table 3:** Hygienic, sanitation, support, and care characteristics of caregivers of children in the orphanages in Addis Ababa, Ethiopia, 2020 (*n* = 271).

Variables	Frequency	Percent
*Wash hands whenever you feed your child*		
Yes	254	93.7
No	17	6.3

*Wash hands before preparing food*		
Yes	244	90.0
No	27	10.0

*Washed hands after visiting the toilet*		
Yes	236	87.1
No	35	12.9

*What did you use to wash hands after visiting the toilet (n* *=* *236)*		
Water with soap	34	12.6
Only water	202	87.4

*Wash hands after cleaning your child*		
Yes	257	94.8
No	14	5.2

*Make water safer to drink*		
Yes	258	95.2
No	13	4.8

*What do you usually do to make the water safer to drink (n* *=* *268)*		
Boil	4	1.5
Add bleach/chlorine/water guard	264	97.4

*Where do your orphanage dispose of domestic waste*		
Open	2	0.7
Pit	269	99.3

*Is there any support given to the orphanage*		
No	267	98.5
Yes	4	1.5

**Table 4:** Factors associated with stunting among orphan under-five children in Addis Ababa, Ethiopia, 2020 (*n* = 271).

Variable	Stunting		COR (95% CI)	AOR (95% CI)
	Yes (*n* %)	No (*n* %)		
*Age of the child*				
6–11	18 (6.6)	34 (12.54)	0.55 (0.27, 1.12)	0.38 (0.16, 5.01)
12–23	27 (9.96)	51 (18.82)	0.56 (0.30, 1.03)	0.42 (0.22, 3.10)
24–35	35 (12.92)	16 (5.91)	2.29 (1.54, 4.71)	2.15 (0.87, 7.21)
≥36	44 (16.23)	46 (16.97)	1	1

*Age of the caregiver*				
19–35	88 (32.47)	79 (29.15)	2.10 (1.38, 3.50)	1.58 (0.75, 8.31)
36–54	36 (13.28)	68 (25.09)	1	1

*Marital status*				
Unmarried	58 (21.41)	53 (19.56)	1.56 (0.93, 2.46)	2.11 (0.65, 4.34)
Married	66 (24.35)	94 (34.69)	1	1

*Illness in the last two weeks*				
Yes	28 (10.33)	64 (23.95)	1.07 (1.03, 8.22)	2.23 (1.41, 12.73)^*∗*^
No	52 (19.19)	127 (46.86)	1	1

*Meal frequency*				
<3 times	98 (36.16)	92 (33.95)	2.25 (1.41, 4.01)	2.11 (1.58, 7.71)^*∗*^
≥3 times	26 (9.59)	55 (20.3)	1	1

*Vaccination received*				
Yes	52 (19.19)	44 (16.24)	1.70 (1.14, 2.80)	2.86 (2.07, 11.61)^*∗*^
No	72 (26.59)	103 (38.01)	1	1

NB: 1: reference group; COR: crude odds ratio; AOR: adjusted odds ratio; ^*∗*^statistically significant at *P* value <0.05.

**Table 5:** Factors associated with wasting among orphan under-five children in Addis Ababa, Ethiopia, 2020 (*n* = 271).

Variable	Wasting		COR (95% CI)	AOR (95% CI)
	Yes (*n* %)	No (*n* %)		
*Age of the child in months*				
6–11	15 (5.54)	37 (13.65)	1.25 (1.05, 5.81)	1.87 (0.89, 8.56)
12–23	14 (5.17)	64 (23.62)	0.68 (0.27, 8.86)	0.41 (0.21, 7.23)
24–35	11 (4.06)	40 (14.76)	0.859 (0.37, 5.65)	0.89 (0.35, 4.53)
≥36	22 (8.12)	68 (25.09)	1	1

*Duration of stay in a month*				
<5	19 (7.01)	99 (36.53)	2.48 (1.13, 5.44)	2.13 (0.72, 6.78)
≥5	11 (4.01)	142 (52.4)	1	1

*Meal frequency*				
<2 times	17 (6.27)	173 (63.84)	0.51 (0.39, 6.22)	0.32 (0.25, 6.57)
≥3 times	13 (4.8)	68 (25.09)	1	1

*Vaccination received*				
Yes	19	156 (50.2)	1	1
No	15	81 (38.75)	1.52 (1.13, 2.87)	2.04 (1.29, 9.71)^*∗*^

NB: 1: reference group; COR: crude odds ratio; AOR: adjusted odds ratio; ^*∗*^statistically significant at *P* value <0.05.

**Table 6:** Factors associated with underweight among orphan under-five children in Addis Ababa, Ethiopia, 2020 (*n* = 271).

Variable	Underweight		COR (95% CI)	AOR (95% CI)
	Yes (*n* %)	No (*n* %)		
*Age of child in months*				
6–11	16 (5.90)	36 (13.28)	1.66 (0.83, 2.49)	1.45 (0.23, 2.65)
12–23	26 (9.59)	52 (9.19)	1.87 (1.04, 3.73)	1.21
24–35	17 (6.27)	34 (12.55)	1.86 (0.86, 4.04)	1.31
≥36	19 (7.01)	71 (26.20)	1	1

*Illness in the last two weeks*				
Yes	25 (9.23)	67 (24.72)	2.12 (1.42, 7.81)	2.36 (0.89, 9.18)
No	35 (12.92)	144 (53.14)	1	1

*Sex of the child*				
Male	51 (26.57)	112 (41.33)	1.24 (0.64, 16.44)	4.25 (0.84, 17.38)
Female	29 (10.70)	79 (29.15)	1	1

*Water treatment*				
Yes	55 (20.30)	176 (64.94)	1	1
No	15 (5.54)	25 (9.23)	1.92 (0.54, 67.23)	3.01 (0.77, 55.72)

*Dietary diversity score*				
<4	72 (26.57)	108 (39.85)	1.67 (1.16, 4.76)	1.32 (1.16, 12.65)
≥4	26 (9.59)	65 (23.99)	1	1

NB: 1: reference group; COR: crude odds ratio; AOR: adjusted odds ratio; ^*∗*^statistically significant at *P* value <0.05.

## Data Availability

The datasets supporting the conclusions of this article are included within the article.

## Abbreviations

AIDS: Acquired immune deficiency syndrome
CDC: Centers for disease control
DDS: Dietary diversity score
EDHS: Ethiopian Demographic Health Survey
FAO: Food and Agriculture Organization
HAZ: Height for age *Z* score
NCHS: National Center for Health Statistics
OVC: Orphan and vulnerable children
UNICEF: United Nations International Children's Fund
WAZ: Weight for age *Z* score
WHO: World Health Organization
RDA: Recommended dietary allowance.
